# Transcriptional response of cultured porcine intestinal epithelial cells to micro algae extracts in the presence and absence of enterotoxigenic *Escherichia coli*

**DOI:** 10.1186/s12263-019-0632-z

**Published:** 2019-03-19

**Authors:** Marcel Hulst, Rommie van der Weide, Arjan Hoekman, Marinus van Krimpen

**Affiliations:** 1 Wageningen Livestock Research, De Elst 1, 6708 WD Wageningen, The Netherlands; 2 Application Centre for Renewable Resources, Wageningen University & Research, Edelhertweg 1, 8219 PH Lelystad, The Netherlands

**Keywords:** Micro algae, Food/feed additive, Intestinal cells, Gene expression, Enterotoxigenic *Escherichia coli*

## Abstract

**Background:**

Micro algae’s are worldwide considered as an alternative source of proteins in diets for animals and humans. Micro algae also produce an array of biological active substances with potential to induce beneficial and health promoting effects. To better understand the mode of action of micro algae’s when applied as additive in diets, porcine intestinal epithelial cells (IPEC-J2), stressed by enterotoxigenic *Escherichia coli* (ETEC) or under non-stressed conditions, were exposed to micro algae extracts and changes in gene expression were recorded.

**Methods:**

IPEC-J2 cells were exposed for 2 and 6 h to extracts prepared from the biomass of the microalgae *Chlorella vulgaris* (C), *Haematococcus pluvialis* (H), *Spirulina platensis* (S), or a mixture of S*cenedesmus obliques* and *Chlorella sorokiniana* (AM), in the absence and presence of ETEC. Gene expression in cells was measured using porcine “whole genome” microarrays.

**Results:**

The micro algae extracts alone enhanced the expression of a set of genes coding for proteins with biological activity that are secreted from cells. These secreted proteins (hereafter denoted as effector proteins; EPs) may regulate processes like remodelling of the extracellular matrix, activation of an antiviral/bacterial response and oxygen homeostasis in the intestine and periphery. Elevated gene expression of immunostimulatory proteins CCL17, CXCL2, CXCL8 (alias IL8), IFNA, IFNL1, HMOX1, ITGB3, and THBS1 was observed in response to all four extracts in the absence or presence of ETEC. For several of these immunostimulatory proteins no elevated expression was observed when cells were exposed to ETEC alone. Furthermore, all extracts highly stimulated expression of an antisense RNA of the mitochondrial/peroxisome symporter SLC25A21 gene in ETEC-challenged cells. Inhibition of SLC25A21 translation by this antisense RNA may impose a concentration gradient of 2-oxoadipic and 2-oxoglutarate, both metabolites of fatty acid β-oxidation, between the cytoplasm and the interior of these organelles.

**Conclusions:**

Exposure of by ETEC stressed intestinal epithelium cells to micro algae extracts affected “fatty acid β-oxidation”, ATP and reactive oxygen species production and (de) hydroxylation of lysine residues in procollagen chains in these cells. Elevated gene expression of specific EPs and immunostimulatory proteins indicated that micro algae extracts, when used as feed/food additive, can steer an array of metabolic and immunological processes in the intestines of humans and monogastric animals stressed by an enteric bacterial pathogen.

**Electronic supplementary material:**

The online version of this article (10.1186/s12263-019-0632-z) contains supplementary material, which is available to authorized users.

## Background

In the last few decades the potential of micro algae as food/feed additive has been recognised and intensively studied. With respect to protein content and quality, the nutritional value of micro algae is comparable or even better than that of conventional plant proteins or macro algae (seaweed) proteins [1]. Therefore, micro algae’s are worldwide considered as an alternative source of proteins in diets for farm animal and humans. From the estimated number of 30.000 species of micro algae that exist, only a few hundred have been analysed on chemical content, and just a few are produced on an industrial scale. Besides many micro algae’s contain substantial amounts of proteins, they also contain a high concentration of, often unique, biological and chemical substances with potential to induce beneficial and health promoting effects in humans and animals [2–5].

The most researched and applied micro algae species to date are the green algae’s *Chlorella vulgaris* (C) and *Haematococcus pluvialis* (H), and the Cyanobacteria (photosynthetic bacteria) of the genus *Spirulina* (S). Especially substances synthesised by the *Spirulina* cyanobacteria were intensively studied for therapeutic, as well as for prophylactic applications. Substances, or groups of substances derived from micro algae’s that were screened for therapeutic potential comprise lipoproteins, alkaloids, amines, flavonoids, sterols, carotenoids (including xanthophyll’s), essential and non-essential vitamins, omega-3 fatty acids and specific toxic secondary metabolites (toxins) [3, 6]. Dependent on the chemical structure of the substance or group of substances, experimental evidence was provided for cytotoxic, antitumor, antibacterial, antifungal, antiprotozoal, antiviral, immunosuppressive and anti-inflammatory activity, or a combination of these activities [2, 4, 7–9]. With regard to prophylactic effects, the antioxidant activity of carotenoids and flavonoids synthesised by micro algae’s have also been studied intensively [8, 10–15]. The antioxidant activity of xanthophyll’s astaxanthin and fucoxanthin, present in high concentrations in the cell wall of micro algae’s, exceeds that of vitamins with antioxidant properties, like β-carotene, vitamin C, vitamin E [16]. When used as supplement in food, it is assumed that these xanthophyll’s contribute to the prevention of oxidative-stress related autoimmune diseases (e.g. atherosclerosis and rheumatoid arthritis) [8].

Many studies showed that micro algae biomass in diets of farm animals can positively change the physiology of animals and improve overall performance of these animals. Besides the above mentioned antimicrobial effects, it was reported that inclusion of micro algae biomass in diets of cattle and poultry positively affected the immune response in these animals, and thereby, improved their resistance against diseases, the function of their intestines, often coincided with an improved feed conversion ratio of these animals as well. However, it has to be noted that inclusion of micro algae biomass in the diets of farm animals not always induced a positive effect on performance. An extended overview of intervention studies in which farm animals were fed with diets supplemented with micro algae preparations is provided in a public accessible report, “Opportunities for micro algae as ingredient in animal diets” [17].

Enterocytes are the predominant cells lined up in the intestinal epithelial layer. They are responsible for absorption of nutrients from the lumen, and for transport of these nutrients over the epithelial layer into the blood stream. Enterocytes and other types of cells in the epithelial layer are covered by a mucus layer, which functions as the first line of defence in preventing harmful microbes and toxic substances to infiltrate underlying tissues and the blood stream. Besides this absorption and barrier function, enterocytes also play a crucial role in the local immune response. Together with specialised immune cells, enterocytes constantly survey the luminal environment for hostile microbes and toxic residues present in the feed/food or formed after digestion of feed/food [18]. In case “danger signals” are sensed, enterocytes transmit signals to underlying cells/tissues and to the body to activate the innate immune defence as well as adaptive immune mechanisms. Enterocytes also sense and transmit non-hostile (beneficial) signals in order to activate an adequate physiological response in the intestines (or elsewhere in the body) to adapt to changes sensed in the lumen (e.g. secretion of digestive enzymes into the lumen and regulation of intestinal motility) [18]. Transmission of signals by enterocytes into the interior of the intestine, or to the body, is performed by “effector proteins” (EP) and chemicals secreted by enterocytes, i.e. enzymes, products and substrates of enzyme reactions, chemicals (e.g. radicals and neuroactive chemicals), hormones, proteins with a specific function (e.g. growth factors), and immune modulator proteins (cytokines and chemokines).

For farm animals, a well characterised porcine intestinal enterocyte cell line (IPEC-J2) can be cultured in the lab and used for gene expression analysis. These cells closely resemble the digestive and absorptive functions of enterocytes in intestine in vivo*,* making them suitable for studying primary interactions of ingredients/additives with enterocytes by gene expression analysis. It was shown that IPEC-J2 cells are able to produce and secrete an array of effector molecules, i.e. cytokines and chemokines (e.g. interleukins, colony stimulating factors, and tumour necrosis factors), several acute phase response proteins, and many other biological active proteins and metabolites, as a response on an exogenous stimulus [19–23]. Compared to human cell lines derived from tumorous colon tissue (e.g. Caco-2, T84, HT-29 and SW620), IPEC-J2 cells are derived from the small intestine, immunologically more responsive, and are not transformed/tumorigenic. In general, tumorigenic cells are less sensitive for stress and cytotoxic compounds and respond differently to exogenous stimuli due to the constitutive expression of a repertoire of “tumour genes”. IPEC-J2 cells mimic the human physiology more closely than any other cell line of non-human origin, and recent studies showed that these cells proved to be a valuable in vitro model for studying primary interactions of food ingredients/additives, pathogens, drugs and toxic compounds with enterocytes for human purposes (reviewed in reference [24]).

When exposed to a pathogenic bacterium like Enterotoxigenic *E. coli* (ETEC) or *Salmonella*, the cytokine/chemokine production by IPEC-J2 cells and cellular stress-processes activated in these cells, largely reflects that of enterocytes in the intestines [20, 21, 23]. However, pathogenic bacteria like ETEC also supresses the expression of cytokines/chemokines in intestinal epithelial cells to avoid an for the pathogens detrimental innate/inflammatory response [25]. An obvious disadvantage of using cultured IPEC-J2 enterocytes for gene expression analysis is the lack of cross-talk between enterocytes, digesta, microbiota, and other types of cells present in the intestines or infiltrating the intestine from the blood flow. Nevertheless, from a welfare and financial point of view, IPEC-J2 cells are an easy to apply and animal friendly tool to measure the immediate-early responses of enterocytes to numerous biological active ingredients/additives and diet formulations [21, 22].

Using gene expression analysis we determined which biological processes and cognate secreted EP’s were activated or silenced in IPEC-J2 enterocytes after exposure to liquid extracts prepared from dried biomass of *Chlorella vulgaris* (C), *Haematococcus pluvialis* (H), *Spirulina platensis* (S) or a mixture of S*cenedesmus obliques*. and *Chlorella sorokiniana* (AM) in the presence, i.e. cells stressed by ETEC bacteria, and in the absence of ETEC. Based on the processes and EPs identified from gene expression data, the biological activity of these micro algae biomasses, when used as feed/food additive, was evaluated.

## Methods

### Preparation of micro algae extracts

Dried biomasses prepared from three micro algae monocultures of *Chlorella vulgaris* strain CCAP 211/11J (C), *Haematococcus pluvialis* strain CCAP 34/7 (H) and *Spirulina platensis* strain SAG 21.99 (S), and from one mixed culture of S*cenedesmus obliques* and *Chlorella sorokiniana* strain SAG:211-8 k (AM), were kindly provided by different suppliers (listed in additional file 1). In additional file 1, the liquid culture media used to grow these micro algae’s and the method of harvesting and drying has been described. Liquid extract were prepared by suspending dried biomass in the culture medium used for growth of IPEC-J2 cells. Technical details about the preparation of extracts are provided in Additional file 1. Briefly, biomass suspensions were desalted by repeated centrifugation and re-suspending of the pelleted biomass, where after the cell walls of the micro algae’s were disrupted mechanically in IPEC-J2 medium using an IKA Ultra-Turrax® Tube Drive homogeniser (Boom B.V Meppel, The Netherlands) with stainless steel and glass beats of different size at 4 °C. After removing the beats and undissolved parts from these extracts by low speed centrifugation, extracts were stored at − 70 °C in aliquots until use. A for IPEC-J2 cells non-toxic concentration was determined for each extracts in a pilot experiment by incubation of serial 10-fold dilutions on IPEC-J2 monolayers.

### IPEC-J2 in vitro test

Micro algae extracts were diluted in culture medium and incubated for 2 and 6 h in 2 cm^2^ culture wells with confluent monolayers of IPEC-J2 cells, in the presence of freshly grown ETEC bacteria (10 colony forming units of per IPEC-J2 cell), or in the absence of ETEC. The *E.coli-*k99 strain with adhesion factor F41 (41/32), isolated from a mastitis-infected udder, was used. Each extract, mixture of extract and ETEC bacteria, and culture medium with ETEC bacteria (without extracts, ETEC alone), were incubated in two independent wells (biological duplicates). After incubation, cells were inspected microscopically where after Trizol reagent (Invitrogen, Thermo Fisher Scientific, Eindhoven, The Netherlands) was added to wells for cell lysis and RNA extraction. Equal amounts of RNA extracted from biological duplicates were pooled and these pools were analysed by gene expression analyses (micro array analysis). Techinical details about the culturing of IPEC-J2 cells and the in vitro test are provided in Additional file 1.

### Gene expression analysis

Briefly, from each RNA pool Cy3 labelled cRNA was synthesised and used to hybridise duplicate patches (technical duplicate) on custom prepared 8x60K Agilent porcine microarrays (Agilent Technologies Netherlands B.V. Amstelveen, The Netherlands). The microarray contains 43,803 oligonucleotide probes representing 28,369 annotated pig mRNAs/genes. Technical details about the preparation of labelled cRNA, hybridisation, scanning of microarrays, generation of data files and the statistical analysis of microarray data are described in Additional file 1. Genes for which a significant higher or lower expression level was measured (differentially expressed genes: hereafter denoted as DEGs) with a *p < 0.05* in IPEC-J2 cells exposed to algae extracts alone or to mixtures of ETEC bacteria and extracts (treated cells) than in cells not exposed to extracts or ETEC (control cells), at the same time point, were selected from data files and listed separately for each comparison. In all results paragraphs beneath information about the biological function of DEGs was retrieved by consulting the “GeneCards” (Weizmann Institute of Science), the NCBI Gene reports (Entrez), and literature linked to these reports (for references about these biological functions of genes we refer to these gene reports). In all tables and additional files official abbreviations for genes are used (HUGO gene-symbols). The raw microarray data are available at the Gene Expression Omnibus (GEO; https://www.ncbi.nlm.nih.gov/geo/) under accession number GSE113365.

### Functional analysis of DEGs

DEGs of each comparison were assigned to a specific pathway/biological process using the bioinformatics program GeneAnalytics (LifeMap Sciences, Inc. USA). Pathways and biological processes significantly enriched in DEGs were retrieved. DEGs coding for an EP, or involved in the metabolism of chemical effector molecules, were selected using the bioinformatics phenotyping program VarElect, which consults information about the function of genes/proteins and related chemicals in the biological databases. EP with a defined role in the above identified “enriched” pathways/processes were further evaluated for their possible role in steering a biological process in the intestines and in the body of animals/humans. In addition, the Comparative Toxicogenomics Database (CTD) was queried to identify curated associations (i.e. based on evidence in the literature) between DEGs and specific (groups of) chemicals.

## Results

### Preparation and toxicity of micro algae extracts

**Fig. 1:**
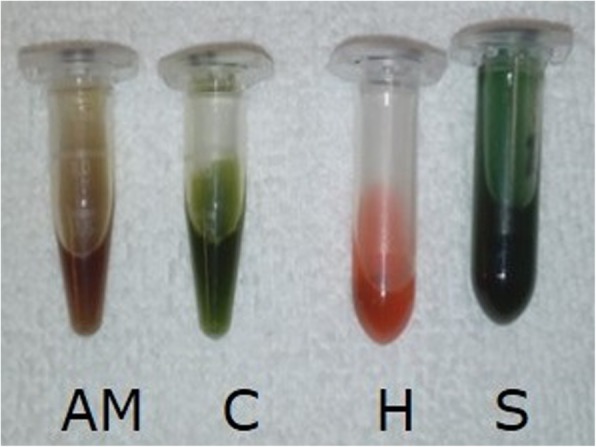
Colour of desalted extracts after mechanical disruption. Note that the colour of the AM extract changed from green to brown due to the orange-red colour of the dye present in the IPEC-J2 culture medium used for desalting

In a pilot experiment, dried biomass of all four micro algae cultures was suspended in IPEC-J2 culture medium to a concentration of 10% (*w*/*v*) and mechanically disrupted using an Ultra-Turrax® Tube Drive with beats (for details see additional file 1). IPEC-J2 cells were incubated with 10-fold dilutions of these extracts for maximum time of 8 h. Inspection of the integrity of the IPEC-J2 monolayer and the morphology of IPEC-J2 cells using a microscope revealed that extracts with concentrations of biomass as low as ~ 0.001% *w*/*v* changed the morphology of the cells drastically and still induced detachment of cells from the monolayer. We anticipated that a high level of salts present in extracts (introduced by the medium in which the micro algae’s were grown) caused this toxic effect on cells. Therefore, fresh suspensions of biomass (10% w/v) were prepared in isotonic IPEC-J2 culture medium and washed twice with this medium by repeated low speed centrifugation and re-suspending of the pelleted biomass. After re-suspending these washed biomasses in IPEC-J2 culture medium, suspensions were homogenised with the Ultra-Turrax® Tube Drive and processed as was done for unwashed extracts. Incubation of IPEC-J2 monolayers for 8 h with dilutions of these desalted extracts showed that this procedure allowed testing of significant less diluted extracts, especially for S and AM (Table 1). Inspection of droplets of extracts using a microscope showed that 50–100% of the micro algae cell walls were broken down by mechanical disruption using the Ultra-Turrax® Tube Drive. For all four extracts an intense coloured solution was obtained in which pigment substances extracted from algae cell walls did not precipitated after centrifugation for 10 min at 200×*g* (see Fig. 1).

**Table 1:** Preparation of non-toxic extracts from micro algae biomass

Micro algae biomass	Untreated	Desalted
	% (*w*/*v*)*	% (w/v)*
C	0.1	1.0
H	0.1	0.2
S	0.001	0.04
AM	0.001	1.0

*maximum concentration of micro algae biomass (gram/100 ml) from which an extract could be prepared that was non-toxic for IPECJ2 cells

### Incubation of extracts on IPEC-J2 monolayers and isolation of RNA from cells

Desalted extracts were diluted two-fold in culture medium and incubated for 2 and 6 h in 2 cm^2^ culture wells with IPEC-J2 monolayers in the presence of freshly grown ETEC bacteria (10 colony forming units per IPEC-J2 cell) or in the absence of ETEC. After 2 and 6 h of incubation, cells were inspected microscopically before RNA was isolated. No detachment, clumping, or rounding of cells was observed for all tested conditions during the 2 or 6 h of incubation, indicating that all mixtures of algae extracts and ETEC bacteria did not cause any visible damage to the cells. Also the yield and quality of RNA isolated for all treatments were comparable to that of control cells (without extract or ETEC). Similar amounts of RNA isolated from biological duplicates were pooled and messenger RNAs (mRNA) in these pools were converted to Cy3 labelled cRNAs for hybridisation on microarrays.

### Gene expression analysis

In Table 2 an overview is given of all the conditions tested in IPEC-J2 cells (treatments), hybridised, and compared to control hybridisations recorded from control cells, including the number of unique genes differently expressed (DEGs) for each treatment. DEGs scoring a fold change (FC, ratio treatment over control) of ≥2 (2 h) or ≥ 3 (6 h) higher (up-regulation) or lower (down-regulation) and scoring a *p-value* < 0.05 were selected for functional analysis [21, 22, 26]. For comparisons of extracts tested in the presence with ETEC at 2 and 6 h, DEGs that also responded to ETEC alone were only selected for functional analysis (see below) in case a 3-fold higher or lower regulation (FC ≥ 3) was observed than in the comparison of ETEC versus control cells. In this manner, pathways/biological processes and important regulatory genes/proteins could be identified which specifically responded to a combination of ETEC and algae extract and not to ETEC alone. In additional file 2, DEGs selected for functional analysis are listed in a separate Excel-sheet for each microarray comparison with their FC, full names and links of webpages containing information about their function.

**Table 2:** Number of DEGs selected from each comparison of treated IPECJ2 cells versus control cells and used for functional analysis

Microarray comparison^$^ (algae extract vs. control)	2 h (FC^&^)	6 h (FC^&^)
E	130 (> 2)	75 (> 3)
C	107 (> 2)	47 (> 3)
C-E	232 (> 3*)	114 (> 3*)
H	30 (> 2)	96 (> 2)
H-E	409 (> 3*)	100 (> 3*)
S	272 (> 2)	91 (> 2)
S-E	14 (> 3*)	37 (> 3*)
AM	ND	727 (> 3)
AM-E	ND	292 (> 3*)

^$^microarray data of treatments were compared to microarray data recorded from control cells (not challenged with ETEC or with algae extracts) that were harvested at the same time point. E = ETEC

^&^FC = fold change cut-off for selection of significantly (*p < 0.05*) differential expressed genes (ratio treatment over control cells)

*DEGs were included in lists for functional analysis in case the measured FC for the comparison of a mixture (algae extract plus ETEC) versus control cells was at least 3-fold higher or lower than measured in the comparison of “ETEC versus control cells” (E) at the same time point

After exposure for 2 h the number of genes regulated in the absence of ETEC for the C (107) and S (272) extracts was substantial higher than observed for the H (30) extract. However, the response to H increased between 2 and 6 h (96), whereas the response to C (47) and S (96) decreased. This suggested that the IPEC-J2 cells adapted to the changed environment imposed by the C and S extracts. After 6 h of exposure, the extract prepared from a mixture of algae’s (AM) was the most potent extract in regulation of gene expression in IPEC-J2 cells (727).

After 2 and 6 h of exposure to ETEC alone, IPEC-J2 cells responded as expected [20, 21, 23]. In the presence of a mixture of ETEC and algae extract for C and H much more changes in gene expression were observed in IPEC-J2 cells compared to cells incubated with ETEC alone. In part, this increase may be due to regulation of gene expression by both ETEC and the biological active substances present in the extracts. However, in case of the H extract, for which only 30 DEGs were measured in the absence of ETEC and much more (409) in the presence of ETEC at 2 h, this increase indicated that biological substances in algae’ extract potentiated the response of IPEC-J2 cells to a challenge with ETEC bacteria.

### Functional analysis of list of DEGs

For lists of DEGs obtained from microarray comparisons, enriched pathways were retrieved using the GeneAnalytics program. In additional file 2 separate lists (Excel-sheets) were prepared for comparisons with and without ETEC. Based on their function, all related pathways were classified into a group representing a dominant biological process (e.g. “inflammation” or “oxidative stress”) and genes/proteins coding for secreted effector proteins (EPs), or having an important regulatory function in these processes, were identified. In Table 3 (without ETEC) and in Table 4 (with ETEC) the results of this functional analysis is displayed.

**Table 3:** Dominant biological processes regulated by algae extracts in the absence of ETEC

Dominant biological processes ^$^	^&^C-2 h	C-6 h	H-2 h	H-6 h	S-2 h	S-6 h	AM-6 h	important DEGs*
androgen receptor signalling					x		x	ITGAV, YWHAH, CSNK2B, CTNNB1,ITGB5
angiogenesis (blood vessel growth)	x	x	x	x			x	HMOX1, FLT1, THBS1, ITGB3, CCL2, NRP2,ITGA6, ITGAV, PTGS2
apoptosis (programmed cell-death)			x		x	x	x	SPTLC1, ITGB3, THBS1, DDIT4, YWHAH
cell motility	x			x	x		x	ITGB3, ANXA1, ITGA6, ITGAV, TPM1, TPM4
cytoskeleton	x			x	x		x	SOS2, PFN2, ARPC1A, ARPC1B, PSEN2, ARPC4, RTN4, ITGB3
extra cellular matrix structure	x	x	x		x		x	CUL1, YWHAH, BTRC /Collagens (COL), SPARC
glycogenesis/glycan synthesis		x		x		x		MUC13, MUC4, HSPG2, PCK2
heat shock (stress)		x					x	DNAJB1, and several heat shock proteins (HSP’s)
hormone processes (Thyroid)					x		x	IGF1R, ITGB3, ITGAV
immune modulation				x		x	x	cytokines, TGF, chemokines
inflammation control	x	x		x	x	x	x	HBEGF, CSF2, CXCL2, IL1A, BIRC3, MMP9, CCL20, LTB, BTRC, HSPB2
neutrophil function (innate immune cells)							x	SOS2, LTBP4, MAPK14, FLT1, IGF1R, PDGFA, ACE, EGFR, DDIT4, FGFR1, TGFA, BMP1, CCL2, RPS6, NRP2
NFKB immune signalling		x	x			x		PTGS2, NFKBIA, CXCL8, TNFAIP3, BTRC
oxidative stress	x	x	x	x	x	x		HMOX1, NQO1, DNAJB1, HSP1A/B
Transforming Growth Factor (TGF)							x	CUL1, THBS1, BTRC, ITGB3
translation (protein synthesis)	x		x		x	x	x	EEF1A1, HSP90B1, DDIT3
tumour necrosis factor (inflammation)		x		x	x	x	x	JAG1, MAPK14, CSF2, CXCL2,TNFAIP3, BIRC3, CASP10, CCL20, CCL2
lipid and steriod metabolism		x					x	SREBF2

^$^Enriched pathways were evaluated and grouped under a dominant biological process

*official Gene-symbols (HUGO abbreviations) are listed for DEGs and DEGs coding for secreted EP’s are underlined. For full names and information about the function of DEGs we refer to the GENECARDS database (web links for each DEG are provided in additional file 2)

^&^Microarray comparison of algae extract (C, H, S or AM) versus control cells at 2 or 6 h

**Table 4:** Dominant biological processes regulated by algae extracts in the presence of ETEC

Dominant biological processes ^$^	E-2 h	E-6 h	^&^C-E2h	C-E6h	H-E2h	H-E6h	S-E2h	S-E6h	AM- E6h	important DEGs*
androgen receptor signalling					x					ITGAV, YWHAH, CSNK2B, CTNNB1, ITGB5
angiogenesis (blood vessel growth)			x	x		x		x	x	HMOX1, FLT1, THBS1, ITGB3, CCL2, NRP2,ITGA6, ITGAV, PTGS2
antigen recognition		x	x			x	x		x	NFKBIE, CXCL2, BCL10, IL1A, FOS, TNFAIP3, BIRC3, NFKBIA, CXCL8
apoptosis	x	x	x		x			x	x	SPTLC1, ITGB3, THBS1, DDIT4, YWHAH
cell motility	x		x		x			x	x	ITGB3, ANXA1, ITGA6, ITGAV, TPM1, TPM4
cytoskeleton					x				x	SOS2, PFN2, ARPC1A, ARPC1B, PSEN2, ARPC4, RTN4, ITGB3
extra cellular matrix structure	x	x	x	x		x			x	CUL1, YWHAH, BTRC/Collagens (COL), SPARC
glycogenesis/glycan synthesis					x	x	x			MUC13, MUC4, HSPG2, PCK2
hormone processes (Thyroid)							x			IGF1R, ITGB3, ITGAV
immune modulation		x	x			x	x		x	cytokines, TGF, chemokines
inflammation	x	x	x			x			x	HBEGF, CSF2, CXCL2, IL1A, BIRC3, MMP9, CCL20, LTB, BTRC, HSPB2
neutrophil function (innate immune cells)					x		x			SOS2, LTBP4, MAPK14, FLT1, IGF1R, PDGFA, ACE, EGFR, DDIT4, FGFR1, TGFA, BMP1, CCL2, RPS6, NRP2
NFKB immune signalling		x	x				x			PTGS2, NFKBIA, CXCL8, TNFAIP3, BTRC
oxidative stress		x	x		x	x				HMOX1, NQO1, EGLN3
Transforming Growth Factor	x				x					CUL1, THBS1, BTRC, ITGB3
translation (protein synthesis)	x		x	x	x	x	x	x		EEF1A1, HSP90B1, DDIT3
tumour necrosis factor (inflammation)		x					x	x		JAG1, MAPK14, CSF2, CXCL2, TNFAIP3, BIRC3, CASP10, CCL20, CCL2, NFKBIA

^$^Enriched pathways were evaluated and grouped by their function under a dominant biological process.* official Gene-symbols (HUGO abbreviations) are listed for DEGs and DEGs coding for secreted EP’s are underlined. For full names and information about the function of DEGs we refer to the GENECARDS database (web links for each DEG are provided in additional file 2)

^&^Microarray comparison of a mixture of algae extract (C, H, S, or AM) and ETEC (E) versus control at 2 or 6 h

^#^official gene-symbols (HUGO abbreviations) are listed for DEGs

In the absence of ETEC, most of the processes regulated by the three extracts prepared from monocultures (C, H, and S) of micro algae were also regulated by the extract prepared from the mixed culture (AM) at 6 h. All extracts regulated the expression of several genes coding for cytokines and chemokines and other immune modulating EPs involved in regulation of inflammation and/or activation and attraction of innate (e.g. macrophages and neutrophils) and adaptive (T cells) immune cells. Beside regulation of immune processes, all extracts also regulated the expression of genes coding for extracellular matrix proteins, i.e. integrin’s (ITG’s) and of collagens (COL’s), and of genes involved in protecting tissues and cells from oxidative stress (see also below), and of genes involved in regulation of processes related to the development, function and structure of blood vessels.

The dominant biological processes identified for mixtures of ETEC and extracts were largely similar to that observed for algae extracts alone (compare Table 3 and Table 4). Several of these processes were not identified for ETEC alone (e.g. angiogenesis, neutrophil function, glycogenesis/glycan synthesis).

### DEGs with a regulatory function

From the common biological processes identified for ETEC alone and for mixtures of algae extracts and ETEC, DEGs with the potential to steer these processes (stimulation, normalisation or silencing of a process) were identified by consulting functional information about these genes and the pathways in which these genes act. In Table 5, these regulatory genes are listed with their full names, a brief description of their potential role/function in these biological processes, and the difference in regulation observed for these DEG’s between ETEC alone and mixtures of ETEC and algae extracts. Note that some of these DEGs may also have an effector function (see below).

**Table 5:** DEGs with an important regulatory function in biological processes induced by algae extracts

^#^DEG	full name	function	dominant biological process	identified pathway	regulation by ETEC (h)	regulation by algae extracts in the presence of ETEC
ANXA1	Annexin A1	anti-inflammatory activity / cell polarisation and cell migration	cell motility	Smooth Muscle Contraction	up (2 h)	up-regulated by S alone and slight enhancement of ETEC-induced up-regulation by S-ETEC mixture
BTRC	Beta-Transducin Repeat Containing E3 Ubiquitin Protein Ligase	ubiquitinates phosphorylated NFKBIA, targeting it for degradation and activating nuclear factor kappa-B.	Transforming Growth Factor (TGF)	TGF-beta Receptor Signalling Pathway	down (2 h)	slight enhancement of ETEC-induced down-regulation by C-ETEC and H-ETEC mixtures
ISG15	ISG15 Ubiquitin-Like Modifier	regulation of antiviral response	translation (protein synthesis) and antigen recognition	Influenza Viral RNA Transcription and Replication	up (2 h)	up-regulated by S and S-ETEC mixture and silencing of ETEC-induced up-regulation by C-ETEC and H-ETEC mixtures
MYLK	Myosin Light Chain Kinase	gastrointestinal motility, vascular permeability, leukocyte diapedesis	cell motility	Smooth Muscle Contraction	none (2 h)	down-regulation by C and H was abrogated by C-ETEC and H-ETEC mixtures
PIAS2	Protein Inhibitor Of Activated STAT 2	inhibition of transcription by STAT2 in immune processes	Transforming Growth Factor (TGF)	TGF-beta Receptor Signalling Pathway	none (2 h)	down-regulated by H-ETEC mixture
NFKBIA	NFKB Inhibitor Alpha	inhibition of transcription by NFKB in immune processes	antigen recognition / inflammation	Toll-Like Receptor Signalling Pathways	none (2 h)	up-regulated by mixtures of C-ETEC and H-ETEC
CASP10	Caspase 10	execution-phase of cell apoptosis	tumour necrosis factor (inflammation) and apoptosis	TNFR1 Pathway	none (6 h)	up-regulated by mixtures of C-ETEC, H-ETEC and S-ETEC, and by AM in the presence and absence of ETEC
HMOX1	Heme Oxygenase 1	Cyto-protection effects / blood vessel widening	angiogenesis (blood vessel growth) and oxidative stress	Keap1-Nrf2 Pathway / angiogenesis (CST)	none (6 h)	variable up-regulation by C, H, S, AM in the presence and absence of ETEC
HSPA1A/B	Heat Shock Protein Family A (Hsp70) Member 1A	control of protein folding and degradation	heat shock (stress) and tumour necrosis factor (inflammation)	TNFR1 Pathway	none (6 h)	up-regulated by mixtures of AM-ETEC, C-ETEC and S-ETEC, and by AM in the absence of ETEC
NFKBIE	NFKB Inhibitor Epsilon	inhibition of transcription by NFKB in immune processes	tumour necrosis factor (inflammation)	TNFR1 Pathway	up (6 h)	silencing of ETEC-induced up-regulation by AM-ETEC, C-ETEC, and H-ETEC mixtures
TNFAIP3	TNF Alpha Induced Protein 3	termination of NF-kappa-B activity and production of inflammatory cytokines	tumour necrosis factor (inflammation) and apoptosis	TNFR1 Pathway	up (6 h)	silencing of ETEC-induced up-regulation by AM-ETEC and C-ETEC mixtures
SPARC	Secreted Protein Acidic And Cysteine Rich	extracellular matrix synthesis and regulation of cell shape	extra cellular matrix structure	Cell Adhesion / ECM Remodelling	none (6 h)	down-regulation by AM alone and by AM-ETEC and C-ETEC mixture

^#^ official gene-symbols (HUGO abbreviations) are listed for DEGs

### Associations of (immune) EPs with biological substances produced by micro algae

**Fig. 2:**
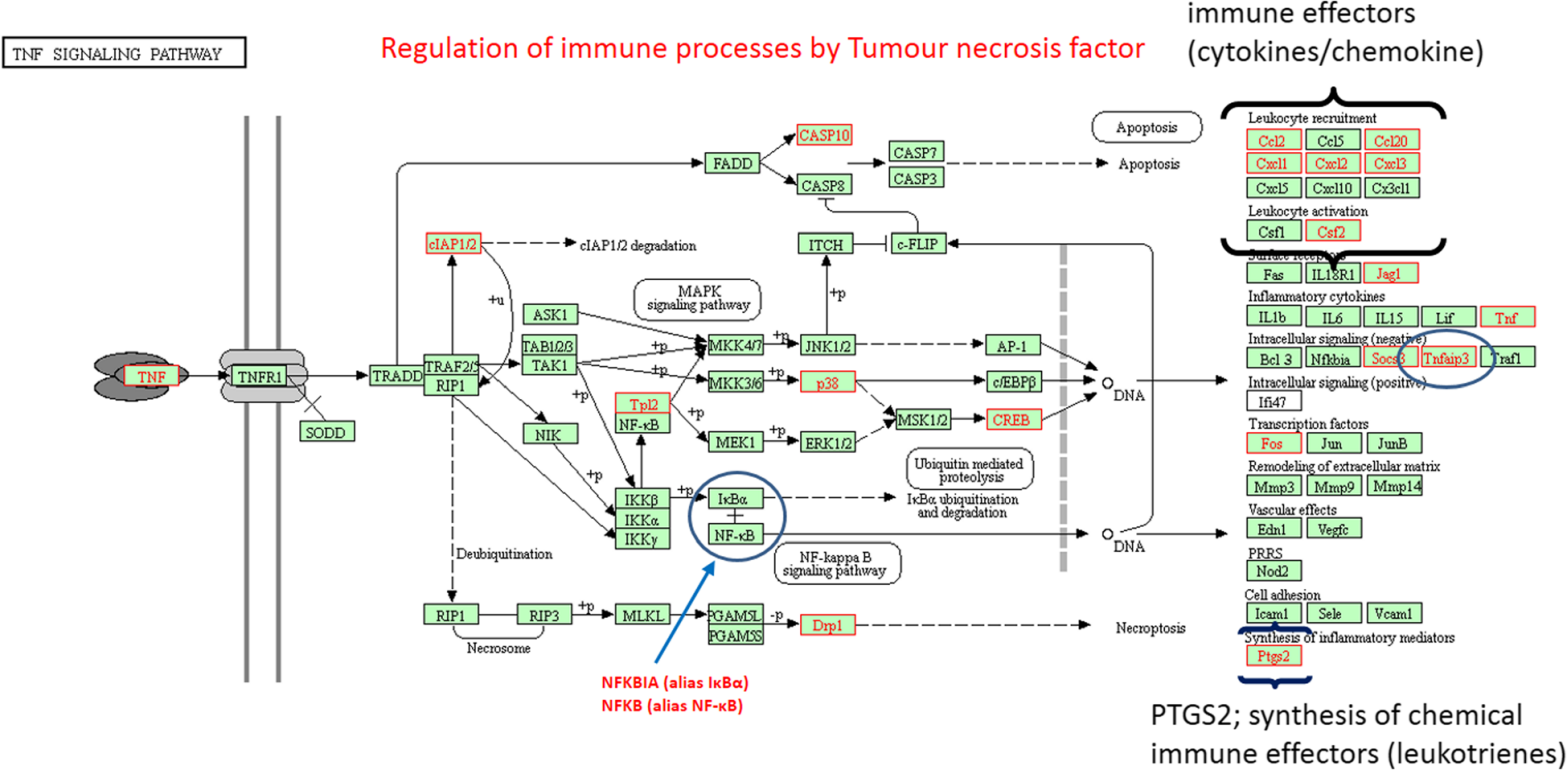
Regulation of immune EP expression by Tumour necrosis factor (TNF) signalling after exposure of IPEC-J2 cells to micro algae extracts in the absence and presence of ETEC. DEGs are highlighted in red or encircled in the original KEGG “TNF signalling pathway” scheme

A sub list of genes coding for secreted proteins with cytokine/chemokine activity or essential for the metabolism of immune modulating chemicals were extracted from all DEGs mapped to enriched pathways/biological processes. In Additional file 3 a complete list of these immune EP’s, with their full names, and FC measured in each microarray comparison, is provided in a separate sheet. In Table 6. the full names of 7 immune EP’s, all higher expressed in IPEC-J2 cells in response to algae extracts alone and to mixtures of extracts and ETEC (but not after exposure to ETEC alone), were listed with a brief description of their function in immunological processes. For this set of common EPs, the CTD was consulted to retrieve associations with biological active substances known to be present in micro algae extracts (Table 6). In Fig. 2 a scheme of the Tumour necrosis factor (TNF) signalling, a pathway that activates inflammatory processes through steering transcription of cytokines/chemokine genes and of genes coding for other immune modulators. In this scheme, DEGs coding for proteins that stimulate or inhibit of TNF-mediated transcription (e.g. NFKBIA/E, NFKB and TNFAIP3) were also highlighted.

**Table 6:** Secreted immune EPs higher expressed in IPECJ2 cells upon exposure to micro algae extracts alone and mixtures of extracts and ETEC

^#^DEG	Full Name	function	association with chemicals produced by microalgae
CCL17	C-C Motif Chemokine Ligand 17	T cell development in thymus and attraction and activation of mature T cells.	carotenoids / flavonoids
CXCL2	C-X-C Motif Chemokine Ligand 2	leukocyte recruitment (including T-cells)	carotenoids / flavonoids /endotoxins / sulphated polysaccharides (Carrageenan)
CXCL8	C-X-C Motif Chemokine Ligand 8	major mediator inflammatory response / attraction and activation neutrophils and macrophages	Fatty Acids, Omega-3 / carotenoids / flavonoids / Phytosterols (e.g. coagulin-L) / sulphated polysaccharides (Carrageenan)
IFNA1	Interferon Alpha 1	cytokine mediating an antiviral state	micro algae lectins
IFNL1	Interferon Lambda 1	cytokine mediating an antiviral state	flavonoids
HMOX1	Heme Oxygenase 1	protection against oxidative stress (breakdown of apoptosis-inducing free heme) / blood vessel widening (vasodilation).	Fatty Acids, Omega-3 / carotenoids (astaxanthine) / flavonoids / Phytosterols (e.g. coagulin-L) / sulphated polysaccharides (e.g. fucoidan)
ITGB3	Integrin Subunit Beta 3 receptor	thrombospondin and metallo-protease mediated ECM remodelling and focal adhesion (e.g. in the process of trans-endothelial migration of leukocytes)	flavonoids
THBS1	Thrombospondin 1	glycoprotein mediating cell-to-cell and cell-to-matrix interactions /anti-angiogenic properties/ fibrotic response in inflammation	Fatty Acids, Omega-3 / carotenoids / flavonoids / Phytosterols / sulphated polysaccharides

^#^official gene-symbols (HUGO abbreviations) are listed for DEGs

Four EPs that were higher expressed after exposure of IPEC-J2 cells to all 4 algae extracts at 6 h, but not after exposure to ETEC alone, were mapped to NRF2 pathway: i.e. HMOX1, NQO1, and heat shock proteins HSPA1A/B (alias Hsp70) and DNAJB1 (alias Hsp40) (see Table 3). The NRF2 pathway represents the activation of NFE2L2 (alias NRF2), a transcription factor responding to oxidative stress in cells. NFE2L2 drives transcription of the above described four DEGs and of many other antioxidant genes (https://www.wikipathways.org/index.php/Pathway:WP2884). Clear associations were found between HMOX1, NQO1 and HSPA1A/B and chemical compounds with antioxidant activity known to be present in micro algae’s (e.g. Tocopherols [Vitamin E and α-Tocopherol], Thioctic acid [alias α-liopic acid; a precursor for ω-3 polyunsatured fatty acids, and Vitamin C [27]) were observed. A complete list of these substances is provided in Additional file 3 (sheet NRF2 pathway).

### Response of ECM genes/proteins

**Fig. 3:**
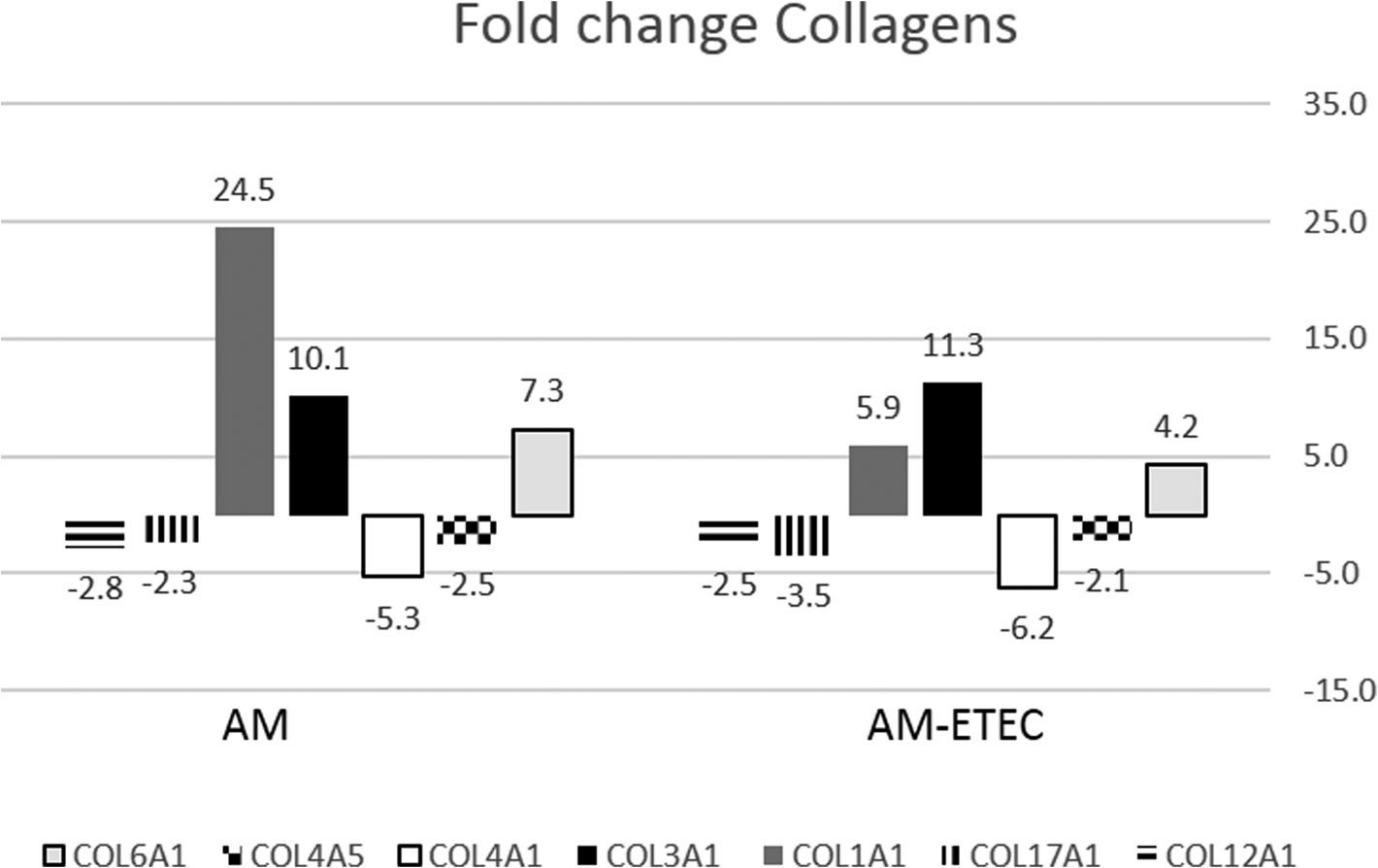
Gene expression pattern of collagen variants in IPEC-J2 cells in response to the AM extract, with and without ETEC

Several genes coding for different types of collagens (COLs) were differentially expressed in IPEC-J2 cells exposed to algae extracts alone and in the presence of ETEC. The C (2 h), H (6 h) and AM (6 h) were the most potent extracts in steering expressing of these genes. An nearly similar pattern of expression of different COL variants was observed for the AM extract alone as for a mixture of this extract and ETEC (Fig. 3), i.e. stimulated expression of the variants COL1A1, COL3A1, and COL6A1, and a decreased expression for COL4A1, COL4A5, COL12A1 and COL17A1. COL3A1 is highly expressed in granulation tissue, new tissue marbled with microscopic blood vessels, whereas COL1A1 and COL6A1 are ubiquitous expressed in all organs including smooth muscle tissue imbedded in blood vessel walls. In the presence of ETEC, gene expression of PLOD2 (an enzyme which reversibly catalyses the hydroxylation of lysine residues in Procollagen) and of COLGALT1 (a Procollagen galactosyltransferase) were down-regulated by the C extract at 2 h and by C and AM at 6 h, respectively. Expression of Integrin Subunit Alpha V (ITGV), a gene coding for a surface receptor that reverses the process of cell-cell adhesion (focal adhesion) was significantly decreased by the AM (6 h) and C (2 and 6 h) extracts. Furthermore, expression of the EP SPARC (secreted protein acidic and rich in cysteine), a stress-inducible protein that plays an important role in disassembly and repair of ECM networks, was decreased by the AM, and C extracts at 6 h, in the absence and presence of ETEC. Elevated expression induced by ETEC alone (FC = 47 at 6 h) of the secreted Alpha-1-Microglobulin gene (AMBP; alias Bikunin Precursor), a serine protease inhibitor exerting an inhibitory activity towards ECM degrading enzymes Elastase-2 (ELANE) and Plasmin (PLG), was further increased by the C, H and AM extracts at 6 h (~ 600-fold for the H and AM extract).

### Genes involved in β-oxidation of fatty acids

**Fig. 4:**
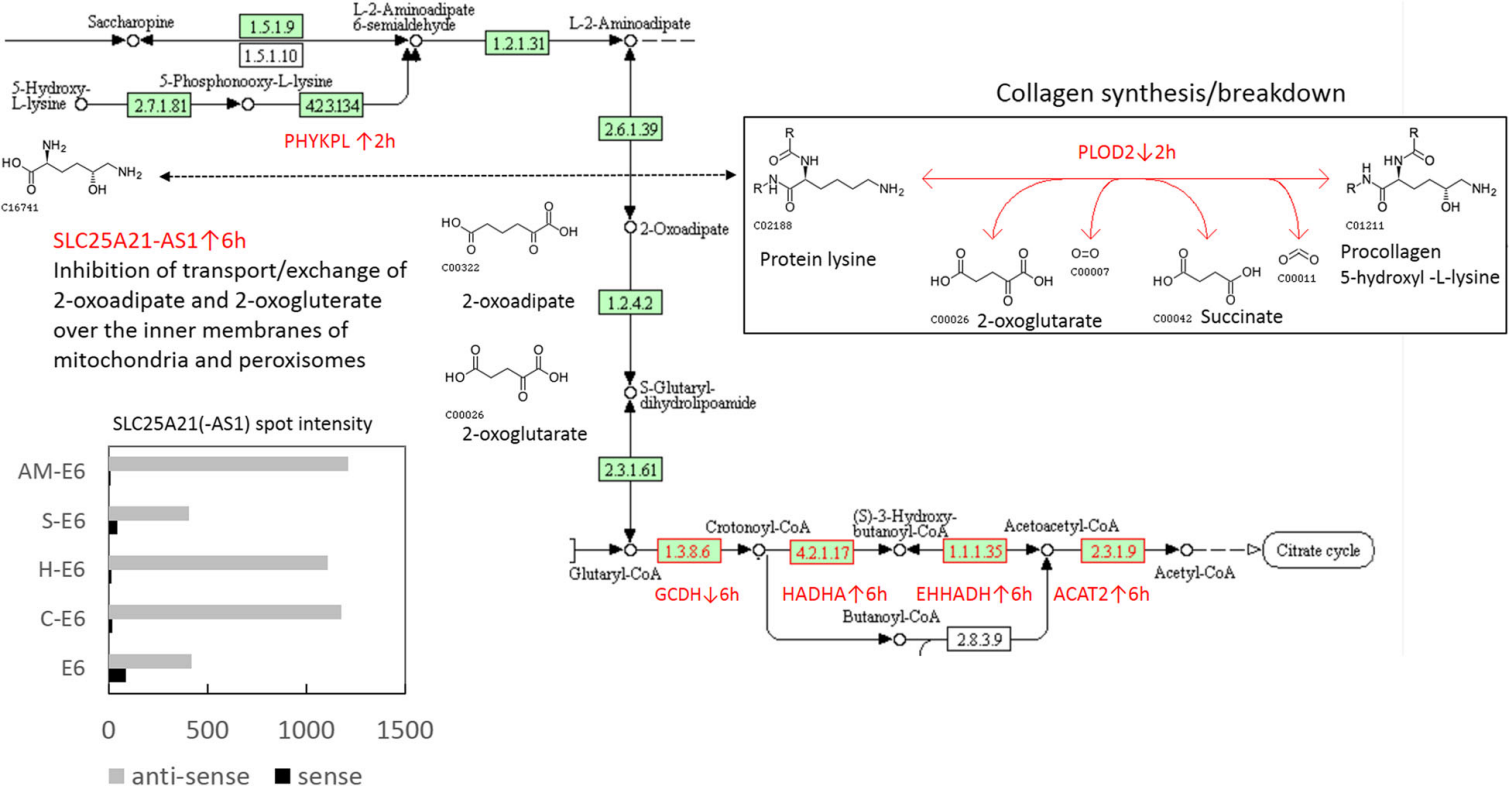
Regulation of Acetyl-CoA production by β-oxidation of fatty acids in mitochondria and peroxisomes, and the interexchange of 2-oxodipate and 2-oxogluterate by SLC25A21 over the membrane of these organelles in cells. DEGs coding for enzymes involved in β-oxidation and in the catabolism of Procollagen 5-hydroxy-L-lysine residues that responded to mixtures of algae extracts and ETEC are highlighted in red in the original KEGG “Lysine degradation” pathway scheme. Graph: raw spot intensity of SLC25A21 (sense) and SLC2521-AS1 (anti-sense) probes on the micro array

The nucleic acid phenotyping program VarElect was used to select genes involved in β-oxidation of fatty acids in mitochondria and peroxisomes from list with DEGs. In the absence of ETEC at 2 h only a few genes could be retrieved matching the queried term “beta-oxidation”. In the presence of ETEC, most DEGs could be retrieved from lists of C and H at 2 h (18), and from lists of AM and C at 6 h (22). These 18 and 22 DEGs were analysed separately using GeneAnalytics in order to retrieve common and related pathways (pathways listed in Additional file 3, sheet Beta-oxidation). Among the pathways retrieved were ″PPAR-mediated transcription of genes involved in membrane transport of fatty acids (DBI, ACSL1 and ACSL4) and the production of Acyl/Acetyl-CoA from fatty acids in mitochondria (ACOX1, CPT1A, ACADM). Two co-activators of PPAR-γ were up-regulated (FAM120A and FAM120C) by the C and H extract in the presence of ETEC at 2 h. In addition to mitochondrial enzymes, also genes coding for peroxisome-specific enzymes involved in β-oxidation (EHHADH, ECH1, ABCD3) and in the biogenesis of these organelles (ABCD3 and PEX19) were enriched at 6 h. Several of these mitochondrial and peroxisome enzymes also play a role in the degradation of the essential amino acids valine, leucine, isoleucine, tryptophan and lysine (see also below). In the process of lysine degradation, the fatty acid intermediate 2-oxoadipic acid (alias 2-oxo-hexanedioic acid) is converted by β-oxidation to acetyl coenzyme A (Acetyl-CoA) for production of energy (ATPs) in the citrate cycle. DEGs coding for enzymes involved in β-oxidation and in the catabolism of Procollagen 5-hydroxy-L-lysine residues (see below) were highlighted in red in Fig. 4.

### Highly elevated expression of an anti-sense RNA in response to algae extracts

Expression of an antisense transcript, SLC25A21-AS1, was moderately (5 to11-fold) stimulated by ETEC and by the AM extract alone at 6 h. However, when ETEC-stressed IPEC-J2 cells were exposed to algae extracts, expression of this antisense RNA increased (up to 300–fold) for all 4 extracts (see Additional file 3, sheet beta-oxidation). Natural antisense transcripts (NATs) block the translation of their cognate sense mRNAs to a functional protein, here SLC25A21 (alias ODC). With respect to sense SLC25A21 mRNA, only for the incubation of ETEC (without algae extract) a raw spot intensity just above the background intensity was measured on microarrays (see bar plot in Fig. 4), revealing that hardly any sense SLC25A21 mRNA was detected in all extracted RNA samples (see bar plot in Fig. 4). SLC25A21 transports C5-C7 oxo-dicarboxylates (fatty acids) across the inner mitochondrial and peroxisome membranes. 2-oxoadipic acid molecules are imported in these organelles in exchange for export of 2-oxoglutarate molecules to the cytoplasm (a so-called membrane symporter). Besides being an intermediate of β-oxidation, 2-oxoglutarate is also converted to succinate as by-product when lysine residues are hydroxylated to 5-hydroxyl-L-lysine residues in Procollagens, catalysed by the PLOD2 enzyme (see above, and the box “Procollagen synthesis/breakdown” in Fig. 4). Expression of the plasma membrane transporter SLC7A11 coincided with SLC25A21-AS1 up-regulation (see additional file 3, sheet Beta-oxidation). SLC7A11 is a subunit of the symporter complex “System Xc-”, which facilitates import of cysteine’s in exchange for export of glutamate molecules in the process of Ferroptosis, a form of reactive oxygen species (ROS) induced cell death. Ferroptosis is triggered by accumulation of iron or Coenzyme A (CoA), the end product of lipid oxidation/degradation. Stimulation of ROS production by eicosanoids synthesised from CoA in the process of Ferroptosis is inhibited by α-Tocopherol (Vitamin E; see KEGG pathway scheme Ferroptosis: http://www.kegg.jp/kegg-bin/show_pathway?map04216+C00010).

## Discussion

To measure the response of IPEC-J2 enterocytes to biological active components produced by micro algae’s it was necessary to desalt the dried biomass by repeated washing with an isotonic medium. Desalting enabled us to test more concentrated micro algae extracts, without toxic effects for IPEC-J2 cells. We observed differential expressed genes involved in steering the processes of blood coagulation and blood vessel development. Both processes are believed to be affected by water-soluble sulphated-polysaccharides produces by algae’s [4, 5, 28]. This suggested that these hydrophilic (water-soluble) substances were, in part, still present after desalting of the biomass in the watery phase. The elevated expression of genes/proteins like HMOX1, NQO1 and CXCL8 induced by algae extracts in the absence of ETEC are believed to be affected by hydrophobic biological active substances produced by micro algae’s (e.g. carotenoids, phytosterols, and ω-3 fatty acids) [29]. Together with the observed intense colouring of extracts after mechanical disruption and centrifugation of algae suspensions, this elevated expression of specific genes indicated that the hydrophobic carotenoid pigments were efficiently extracted from cell walls and stayed biologically active.

When applied as additive in diets, algae’s pass the stomach and the upper parts of the intestines before reaching the enterocytes in the jejunum. In these parts, algae’s are exposed to low pH and to host and microbiota enzymes, resulting in the release of macromolecules fragments like peptides and oligosaccharide moieties from the cells walls. The biological activity of such macromolecule fragments was demonstrated in several studies (reviewed in [7, 11, 30, 31]). Recent research in our lab showed that beat milling of algae biomass improved digestibility of proteins with 10% (van Krimpen, personal communication), indicating that proteins imbedded in the algae cell wall matrix were fragmentised and unlocked by this method, a method comparable to the Ultra-Turrax® disruption method with beats we used in this study. This suggests that our extracts may also contain soluble peptides and oligosaccharide moieties released from cell walls, which may have contributed to the overall response measured in IPEC-J2 cells. Because, mechanical disruption, most likely, generates a randomised population of moieties with unknown structure, we are not able to relate the responses in IPEC-J2 cells to moieties with a specific chemical structure, as was established for anticoagulant activity of specific sulphated glycan motifs [32] and antibacterial activity of peptides and proteinaceous lectins [7, 9]. Another indication that biological active substances were efficiently extracted from the C, H, S and AM biomass was the overlap in biological processes regulated by all four extracts in not stressed, as well as in and ETEC-stressed IPEC-J2 enterocytes. The extracts prepared from the mixed culture (AM) was the most potent one, likely, because it contained a broader collection of biological active substances.

### Activation of immunostimulatory EPs

Expression of genes coding for cytokines/chemokines (CXCL8, CSF2, CCL2, CCL5, CCL17, CCL20,) that attract and activate innate immune cells (e.g. macrophages, neutrophils) and cells of the adaptive immune response (T- and B-cells), was stimulated by the tested micro algae extracts. Berry et al. [33] recently showed that expression of CCL20 and CXCL8 increased when IPEC-1 cells (a cell line closely related to IPEC-J2) were exposed to purified fractions of sulphated polysaccharides prepared from seaweed. The elevated expression of an array of cytokines/chemokines suggests that our extracts, most likely, also contain other biological substances than sulphated polysaccharides that are able to directly or indirectly activate expression of a variety of functional different chemokines/cytokines in IPEC-J2 cells. This (immediate)-early immunological response we measured in IPEC-J2 cells will be useful for designing and conducting in vivo intervention studies addressing the immunological potential of micro algae extracts or specific biological substances purified from micro algae’s for humans and animal intestinal health purposes.

In several studies it was reported that extracts of macro and micro algae supressed the expression of the inflammatory cytokines TNF-α, TGF, IL1B, IL6, IL4, IFNG [12, 34–36] and of the transcription factor NFKB responsible for expression of these cytokines [37]. We also observed a lower expression of the cytokines TNF-α (S), TGF-α and TGF β (AM) in IPEC-J2 cell. In addition, after exposure of IPEC-J2 cells to mixtures of ETEC and algae extracts for 2 h, all extracts stimulated the expression of NFKBIA, the inhibitor of NFKB transcription activity (see Fig. 2), and of TNFAIP3, a terminator of TNF signalling (blue encircled in Fig. 2). At this time-point, expression of NFKBIA and TNFAIP3 was not stimulated by ETEC or extracts alone. This possible feed-back response by NFKBIA and TNFAIP3 could play a role in tuning expression of inflammatory cytokines by IPEC-J2 cells, i.e. to prevent overexpression.

Expression of COL1A (Type I Procollagen Alpha 1 Chain) was highly stimulated by the AM extract alone (FC ~ 25). Overexpression of procollagens in inflamed tissue leads to formation of fibrils that initiate inflammatory processes. Procollagen overexpression is presumed to be associated with increased expression of the heat shock protein (HSP) and pro-collagen chaperone, SERPINH1 (alias Colligin-1 or HSP [38, 39]). However, we observed no elevated gene expression of SERPINH1 in response to tested extracts, whereas we did for two other HSP’s (DNAJB and HSPA1A/B; alias HSP70.1). In IPEC-J2 cells challenged with the AM extract alone, expression of these two HSP’s coincided with the elevated COL1A expression. In the intestinal mucosa of patients with inflammatory bowel disease, HSPA1A/B overexpression was found to be associated with a balanced inflammatory response to damaging factors [40]. It would be interesting to investigate if this co-elevated expression of COL1A and HSP’s in enterocytes also play a role in controlling inflammatory reactions in vivo. Furthermore, our data suggest that high concentrations of algae biomass in a diet could trigger the formation of “inflammatory fibrils”, and consequently unwanted inflammatory reactions. Therefore, we propose that dose-response studies have to be performed in order to define a concentration of micro algae biomass in a diet which does not induce negative side-effects, but still supports the beneficial effects of the biological active substances produced by the micro algae.

### Antiviral properties

Sulphated polysaccharides, specific lectins, and the phlorotannin Diphlorethohydroxycarmalol (a flavonoid derivate), were identified as micro algae substances with antiviral activity [15, 30, 41, 42]. The latter substance inhibited integration of the HIV genome in host cell DNA [43] and stimulated the expression of genes coding for enzymes that synthesise the inflammatory mediating prostaglandins (COX1, COX2 and PTGS2), and of genes involved in the repair of damaged DNA (nucleotide excision repair genes; ERCC’s) [44, 45]. In our study, a lower expression of prostaglandin synthesising enzymes and ERCC’s was measured in response to micro algae extracts, and no other indicative evidence was found that these processes were stimulated in IPEC-J2 cells. Nevertheless, the current study showed that expression of the interferon genes IFNL (alias IL29), IFNW1 (2 h) and IFN1A (6 h) was stimulated by micro algae extracts alone and by mixtures of extracts and ETEC (i.e. in stressed and non-stressed IPEC-J2 cells). Expression of these interferons can be induced via the “RIG-I-like receptor signalling pathway” (https://www.kegg.jp/kegg-bin/show_pathway?hsa04622) when cells sense viral antigens. Further characterisation of the micro algae extracts in order to determine if specific compounds in the extracts are responsible for this stimulated interferon expression, and if such compounds have potential to induce an interferon-induced antiviral response to enteric human and animal viruses, may be worthwhile have potential.

### Antioxidant activity

As reported in other studies [16, 29], all here tested algae extracts highly stimulated expression of HMOX1 at 6 h, in the absence and presence of ETEC. In contrary, no enhanced expression was observed for HMOX1 after 2 and 6 h for ETEC alone. HMOX1 is an enzyme involved in degradation of heme, the oxygen-binding co-factor present in proteins like haemoglobin and myoglobin. By degrading free circulating heme, which induces oxidative-stress in cells, HMOX1 protects cells from programmed cell-death. Together with other antioxidant proteins/enzymes, expression of HMOX1 (and also of NQO1) is initiated by binding of the transcription factor NFE2L2 to the antioxidant response element (ARE) in the DNA loci of these genes. For NFE2L2, a lower expression was observed after exposure of IPEC-J2 cells for 2 h to algae extracts (C and H) in the presence of ETEC. After 6 h under these conditions, expression of NFE2L2 returned to a normal level. Likely, a normal concentration of NFE2L2 was sufficient to support the high expression of the antioxidant gene HMOX1, even in the presence of ETEC. These results, and the stimulation of cytokine expression (see above), indicated that substances in the micro algae extracts were able to exert their biological activity also in enterocytes stressed by an bacterial pathogen like ETEC.

### Oxygen homeostasis

With respect to antioxidant activity we also observed differential expression of several genes coding for enzymes involved in β-oxidation of fatty acids in mitochondria and/or peroxisomes (highlighted in red in Fig. 4). Beta-oxidation of fatty acids produces Acetyl-CoA molecules for ATP (energy) production in the citric acid cycle. Beta-oxidation is regulated by PPAR-signalling and may result in the production of an excess of ROS, and subsequent, to cell damage and programmed cell death [46]. The extremely high elevated expression of antisense RNA SLC25A21-AS1 in ETEC-stressed cells induced by all four micro algae extracts could play an important role in preventing an overload of ROS. As mentioned above, this antisense RNA inhibits translation of the oxodicarboxylate-symporter SLC25A21, affecting the concentration of 2-oxoadipate and 2-Oxoglutarate in the interior of mitochondria/peroxisomes and in the cytoplasm. Changes in 2-oxoglutarate concentration in the cytoplasm are sensed by egl-9 family hypoxia-inducible factors (e.g. EGLN3) which regulates the activity of the transcription factor hypoxia-induced factor 1A (HIF1A). HIF1A is the key transcription factor for the expression of genes responsible for regulation of oxygen homeostasis in cells, i.e. HIF1A regulates the switch from aerobic to anaerobic energy metabolism and vice versa in cells. In addition, HIF1A also stimulates the expression of EPs like HMOX1, a protein that widens blood vessels to support oxygen supply to cells in tissues.

### Collagen synthesis/breakdown

In the hydroxylation reaction of lysine residues in COL chains, catalysed by the PLOD enzymes, 2-oxoglutarate donates an O_2_ molecule and is converted to succinate (see box in Fig. 4). Succinate stabilises HIF1A [47], indicating that differences of 2-oxoadipate and 2-oxoglutarate concentrations between the mitochondria/peroxisomes and the cytoplasm not only affected oxygen homeostasis/ROS production in IPEC-J2 cells, but also the synthesis/breakdown of COLs. However, from our current data we cannot point out which lipophilic substance(s) in algae extracts were responsible for this, and it is not clear whether ROS are neutralised or that ROS production was favoured, e.g. to inhibit colonisation of ETEC bacteria. Also, it is not clear which biological substance, e.g. α-Tocopherol (alias Vitamin E) [48], Astaxanthin [16, 49], ω-3 fatty acids [50], Thioctic acid (alias “pyruvate oxidation factor” or α-lipoic acid, a precursor of ω-3 fatty acids) [27, 51] or combinations of substances in our micro algae extracts induced, e.g. transcription of SLC25A21-AS1, that may play a key role in regulation of these processes.

For the fresh water micro algae *Scenedesmus*, a stimulated expression of COLs, and in particular of COL3A1, was associated with suppression of UV irradiation-induced signs of ageing in skin cells [52]. Also, phospholipids isolated from *Chlorella regularis* affected COL/elastin ratios in the aorta of rats [53]. These results are in line with our data, and indicate that both species in the AM mixture modulate COL synthesis and breakdown. Moreover, it could explain the specific pattern of COL expression we observed, elevated expression of COL variants found in blood vessel walls.

### Blood vessel growth and proliferation

The elevated expression of COL3A1, which is a vital constituents of micro blood vessels, and of genes which promote blood vessel growth (angiogenesis; see Table 4) suggests that the micro algae extracts may promote forming and maintenance of lamina propia tissue in the intestine. The higher expression of the EP HMOX1, which promotes formation and widening of blood vessels in ischemic tissue [54] and the lower expression of the EP SPARC, an EP that inhibits proliferation of endothelial cells in vessel walls [55, 56], are in line with this hypothesis. Remodelling of ECM networks and proliferation of blood vessel cells are processes which also facilitate the passage of leukocytes through vessel walls, a process called diapedesis [57]. Together with the elevated expression of cytokines that attract these leucocytes (see above), this suggests that in vivo micro algae extracts may also potentiate diapedesis by regulating expression and secretion of EPs like HMOX1 and SPARC.

## Conclusions

All four tested micro algae extracts stimulated the expression of an array of cytokines/chemokines, irrespective of the presence of ETEC. These cytokines/chemokines may promote the influx of innate and adaptive immune cells into the lamina propia, enabling the intestinal immune system to respond quickly and properly to toxic compounds and enteric pathogenic intruders like ETEC.

Similar as reported in literature, all four extracts stimulated the expression of antioxidant proteins like HMOX1 and NQO1, in non-stressed and ETEC stressed cells [8, 28, 29]. The strong elevation of expression of the anti-sense transcript of the 2-oxoadipate/2-oxoglutarate symporter SLC25A21 in ETEC-stressed cells, induced by all four algae extracts, suggested that the process of collagen synthesis/breakdown was linked to ATP production via β-oxidation of fatty acids and to oxygen homeostasis/ROS in IPEC-J2 enterocytes. Transcriptional regulation of an anti-sense RNA induced by specific substances produced by micro algae’s, is to our knowledge, an novel finding which needs further research.

## Additional files

Additional file 1: Methods A). Growth of micro algae’s and preparation of dried biomass. B). Morphological typing of algae’s by microscopy. C). Preparation of algae extracts. D). IPEC-J2 in vitro test. E). Labelling, hybridization, scanning and feature extraction of microarrays. (DOCX 1779 kb)

Additional file 2: DEGs microarray comparisons and functional (pathway) analysis. A). Lists of DEGs used for pathway analysis. B). Enriched pathways without ETEC. C). Enriched pathways with ETEC. (XLSX 392 kb)

Additional file 3: DEGs involved in specific biological processes. A). Regulatory genes. B). Immune effector proteins (EPs). C). NRF2-pathway. D). Beta-oxidation. (XLSX 54 kb)

## Abbreviations

AM: Mixture of *Scenedesmus sp.* and *Chlorella sp.*
C: *Chlorella vulgaris*
COLs: Collagens
CTD: Comparative Toxicogenomics Database
DEGs: Differentially expressed genes
EPs: Effector proteins
ETEC: Enterotoxigenic *Escherichia coli*
FC: Fold change
GEO: Gene expression omnibus
H: *Haematococcus pluvialis*
HUGO: Gene Nomenclature and symbols of human gene names in the HGNC database
IL: Interleukin
IPEC-J2: Porcine intestinal epithelial cell line J2
KEGG: Kyoto Encyclopedia of Genes and Genomes
S: *Spirulina platensis*
TNF: Tumour necrosis factor
