# Supplementary material for: Transcriptional response of cultured porcine intestinal epithelial cells to micro algae extracts in the presence and absence of enterotoxigenic Escherichia coli
Source: Genes Nutr. 2019 Mar 19;14:8. doi: 10.1186/s12263-019-0632-z (PMC6423797; doi:10.1186/s12263-019-0632-z)
Supplement: Supplementary file 1 — Methods A). Growth of micro algae’s and preparation of dried biomass. B). Morphological typing of algae’s by microscopy. C). Preparation of algae extracts. D). IPEC-J2 in vitro test. E). Labelling, hybridization, scanning and feature extraction of microarrays. (DOCX 1779 kb) [file 12263_2019_632_MOESM1_ESM.docx]

**Additional file 1** Materials and Methods

**A) Growth of micro algae’s and preparation of dried biomass.**

Algae cultures were grown in liquid media (specified below) at temperatures around 20°C degrees with artificial light and additional CO_2_ in 5-20 litter vessels. After reaching the desired density, suspensions were centrifuged at low speed and the pellets (paste) was dried in thin layers at 40°C to powder (biomass).

-*Spirulina platensis* stain SAG 21.99 was grown in Modified Spirulina Medium; <http://marinebiotechnology.org/images/stories/medios_cultivo/Spirulina.pdf>.

Supplier; Algae Food and Fuel (note that this firm recently went bankrupt) <https://www.sustainableurbandelta.com/algae-food-fuel/?lang=nl>

-*Haematococcus pluvialis* strain CCAP 34/7 was grown in the medium described by Fábregas, et al. (2000) DOI: <https://doi.org/10.1007/s002530051652>.

Supplier; Astaco, <https://www.astaco-technologies.com/nl/>

-*Chlorella vulgaris* strain CCAP 211/11J and the mixture of S*cenedesmus* *obliques* and *Chlorella* *sorokiniana* (strain SAG:211-8k) were grown in 3N BBM basalt bold medium, described in: BISCHOFF, H.W., and BOLD, H.C. 1963.Phycological Studies IV. Some Soil Algae From Enchanted Rock and Related Algal Specie. University of Texas, Austin, 6318: p1 – 95; <http://ccala.butbn.cas.cz/en/bbm-medium>).

Supplier; Chlorella vulgaris strain, Scenedesmus obliques and Chlorella sorokiniana (strain SAG:211-8k) ACRRES, <http://www.acrres.nl/>

**B)** **Morphological typing of algae algae’s by microscopy.**

**
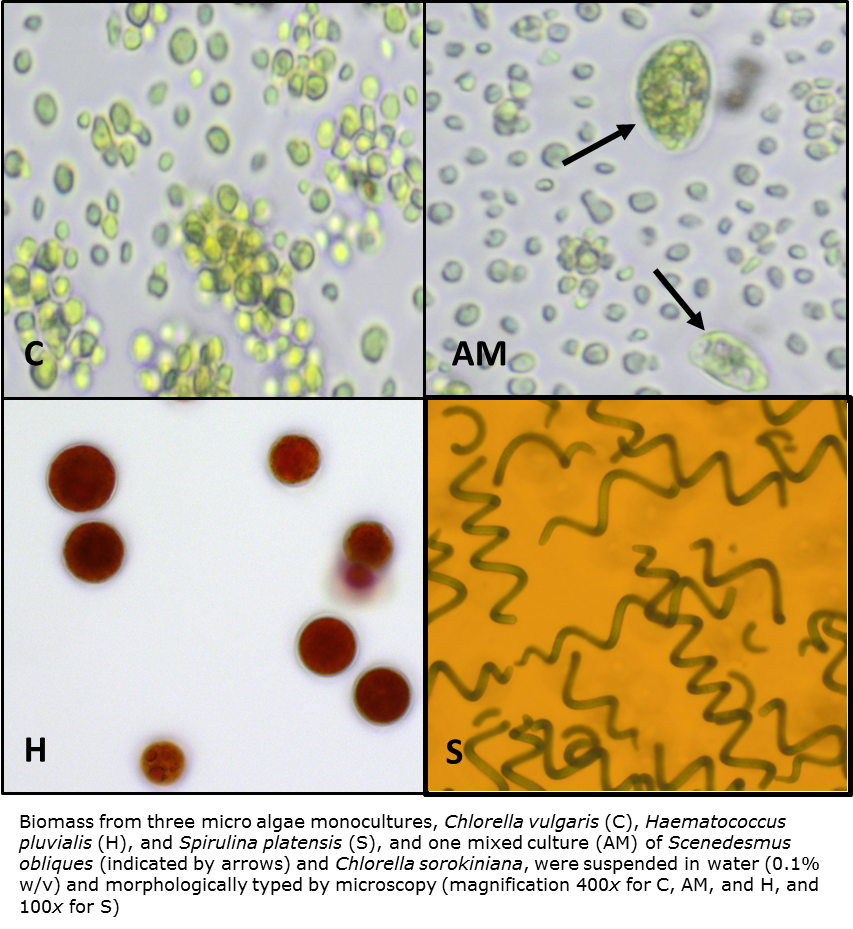
**

**C) Preparation of algae extracts.**

Dried biomass (2.5 g) prepared from micro algae cultures was suspended in 25 ml ice-cold 1:1 DMEM/Ham’s F10 cell culture medium (Gibco/Thermo Fischer scientific, Bleiswijk, The Netherlands) at a concentration of 10% (w/v) and vortexed with glass beats for 15 sec. The suspension was centrifuged for 10 min at 200x*g* and the pellet was re-suspending in 25 ml of ice-cold 1:1 DMEM/Ham’s F10 cell culture medium, centrifuged again, and the pellet was re-suspended in 10 ml of medium. This suspension was homogenised at 4°C for 20 min in an IKA Ultra-Turrax® Tube Drive homogenizer device (IKA, Boom B.V Meppel, The Netherlands) using iron beats and glass beats of different sizes. After placing on ice for 5 min, this homogenisation step was repeated under the same conditions. The homogenate was centrifuged for 10 min at 200x*g* to remove beats and undissolved matter and the supernatant (extract) was harvested and frozen in aliquots at -70°C until use.

**D)** **IPECJ2 in vitro test.**

The effect of extracts prepared from 3 monocultures of micro algae's (C,H, and S) and a mixed culture of micro algae's (AM) was studied in the presence and absence of the enterotoxigenic bacterium Escherichia coli k99 strain (ETEC) as an in vitro challenge. The E.coli-k99 strain with adhesion factor F41 (41/32) was isolated from a mastitis-infected udder. IPECJ2 cells were seeded in 2 cm^2^ tissue culture wells (M24 plates) and grown for 7 days at 37°C and 5% CO2 using 1:1 DMEM/Ham’s F10 medium (Gibco-BRL) supplemented with 5% FCS without antibiotics. For all tests, confluent monolayers were washed twice with medium without FCS (hereafter denoted as medium) and incubated for 1 hour with this medium. Hereafter, the medium was discarded and algae extracts suspended in medium were added to wells with IPECJ2 monolayers and incubated for a period of 2 and 6 hours in the absence and presence of ETEC (±10 cfu per cell). All incubations were tested in duplicate and for each type of extract duplicate control wells containing no additive (only culture medium) or ETEC alone (no extracts) were incubated for 2 and 6 hours. After incubation total RNA was extracted using Trizol (Invitrogen). RNA's extracted from replicate wells were pooled (biological replicates) and each pool was hybridized in duplicate (technical replicates). All the RNA samples scored a RNA integrity number (RIN value) of ≥ 9 (Agilent Lab-on-a-Chip Bioanalyzer).

**E) Labelling, hybridization, scanning and feature extraction of microarrays.**

Briefly, 500 ng RNA of each sample was labelled with the One-Color Microarray-Based Gene Expression Analysis Low input Quick Amp Labelling kit and 600 ng of Cy3 labelled cRNA was used for hybridisation on each patch. Hybridisation and washing of the arrays was performed according to the protocol provided by Agilent Technologies for the One-Color Microarray-Based Gene Expression Analysis Low input Quick Amp Labelling kit. RNA pools were labelled in duplicate and hybridized separately to obtain 2 technical replicates. Arrays were scanned using a DNA microarray scanner with Surescan high resolution Technology (Agilent Technologies) with a resolution of 5 µ, at 16 bits and a PMT of 100%. Feature extraction was performed using protocol 10.7.3.1 (v10.7) for 1 colour gene expression. The files generated by the feature extraction software were loaded in GeneSpring GX 9.0.5, in which a log_2_-transformation and a median normalization (75 percentile) was performed on all probes. Probes with a raw intensity of >60 (flooring) and with a corrected *p-value* of <0.05 (OnewayANOVA significance analysis with asymptotic p-value computation) were selected from the data files, annotated and assigned as differential expressed genes (DEGs) [see reference 22 and 26 in the manuscript].
